# Comparing Device-Generated and Calculated Bioimpedance Variables in Community-Dwelling Older Adults

**DOI:** 10.3390/s24175626

**Published:** 2024-08-30

**Authors:** Kworweinski Lafontant, Danielle A. Sterner, David H. Fukuda, Jeffrey R. Stout, Joon-Hyuk Park, Ladda Thiamwong

**Affiliations:** 1 Institute of Exercise Physiology and Rehabilitation Science, University of Central Florida, Orlando, FL 32816, USA; danielle.sterner@ucf.edu (D.A.S.); david.fukuda@ucf.edu (D.H.F.); jeffrey.stout@ucf.edu (J.R.S.); 2 College of Nursing, University of Central Florida, Orlando, FL 32826, USA; ladda.thiamwong@ucf.edu; 3 Disability Aging & Technology Cluster, University of Central Florida, Orlando, FL 32826, USA; joonpark@ucf.edu; 4 Department of Mechanical and Aerospace Engineering, University of Central Florida, Orlando, FL 32826, USA

**Keywords:** bioimpedance, cellular health, body composition, fitness assessment, InBody

## Abstract

Despite BIA emerging as a clinical tool for assessing older adults, it remains unclear how to calculate whole-body impedance (Z), reactance (Xc), resistance (R), and phase angle (PhA) from segmental values using modern BIA devices that place electrodes on both sides of the body. This investigation aimed to compare both the whole-body and segmental device-generated phase angle (PhA_DG_) with the phase angle calculated using summed Z, Xc, and R from the left, right, and combined sides of the body (PhA_Calc_) and to compare bioelectric variables between sides of the body. A sample of 103 community-dwelling older adults was assessed using a 50 kHz direct segmental multifrequency BIA device. Whole-body PhA_Calc_ values were assessed for agreement with PhA_DG_ using 2.5th and 97.5th quantile nonparametric limits of agreement and Spearman’s rho. Bioelectrical values between sides of the body were compared using Wilcoxon rank and Spearman’s rho. A smaller mean difference was observed between PhA_DG_ and right PhA_Calc_ (−0.004°, *p* = 0.26) than between PhA_Calc_ on the left (0.107°, *p* = 0.01) and on the combined sides (0.107°, *p* < 0.001). The sum of Z, R, and PhA_Calc_ was significantly different (*p* < 0.01) between the left (559.66 ± 99.55 Ω, 556.80 ± 99.52 Ω, 5.51 ± 1.5°, respectively) and the right sides (554.60 ± 94.52 Ω, 552.02 ± 94.23 Ω, 5.41 ± 0.8°, respectively). Bilateral BIA values do not appear to be interchangeable when determining whole-body measurements. Present data suggest that using right-sided segmental values would be the most appropriate choice for calculating whole-body bioelectrical variables.

## 1. Introduction

Bioelectrical impedance analysis (BIA) is a quick, non-invasive, and commercially available method of assessing body composition and cellular health with increasing clinical utility among older adults [1]. BIA works by directing an electrical current through the body from multiple electrodes, commonly placed at the distal ends of the limbs [2,3]. Body composition is estimated based on the two-component model, although other variables can be evaluated using BIA, such as the phase angle (PhA). BIA devices typically calculate PhA at 50 kHz using the reactance (Xc), resistance (R), and impedance (Z) of the electrical current. Xc represents the capacitive effects of cell membranes, while R represents the resistive properties of cells due to fluid distribution [1,4]. Z is the overall opposition to the current, which is derived from Xc and R measures [5]. PhA represents the ratio of Xc and R at a given frequency, defined as the delay in current flow caused by a reduction in cell membrane capacitance [3,4], and it can be calculated using the following equation: [PhA (°) = arc tangent (Xc/R) × (180/π)]. At 50 kHz, PhA is indicative of cellular tissue integrity and can be a sign of possible cell membrane dysfunction [5,6,7]. In older adults, higher PhA values are indicative of superior cell membrane integrity and cellular health, while lower PhA values are associated with sarcopenia and a higher risk of mortality [1,5]. Lower PhA values have also been linked to diabetes, cardiovascular disease, and certain cancers [8,9,10,11], allowing PhA to serve as a clinical marker of global health for older adults and adult populations [12].

Despite the utility of PhA as a clinical marker, it is not uniformly reported by BIA devices. Whether a BIA device reports Z, Xc, R, and PhA at the whole-body level is dependent on the device manufacturer. While a clinician could calculate these variables using their known formulas, doing so would require using segmental values to determine whole-body values [13]. For whole-body PhA, it is unclear which segmental values should be summed to constitute the whole body. The sum of right-sided variables has historically been considered equivalent to a whole-body measure [14]; however, that may be because earlier research on BIA only placed electrodes on the right side of the body, typically at the wrist, shoulder, hip, and ankle [14]. This is contrary to the use of modern BIA devices where electrodes are placed on both sides of the body and shoulder/hip electrodes are typically forgone due to the use of the equipotential method [13,15]. Further, previous research has used bioelectrical values combined from both sides of the body to determine whole-body measures rather than the right side alone [16,17]. There appears to be inconsistency within the scientific community regarding what is considered a whole-body BIA measure, and these inconsistencies further complicate the ability of clinicians to accurately calculate whole-body PhA when it is not reported by the BIA device.

While BIA has great applicability with older adults, research focused on the relationship between segmental and whole-body bioelectrical variables in older adults is sparse. Jensen et al. compared PhA between left and right limbs in 302 adult men and women using a modern BIA device (seca mBCA 514/515, seca GmbH, Hamburg, Germany) with electrodes on both sides of the body and reported no significant differences between the left and right sides [18]. Despite no statistical significance, the PhA on the right side tended to be greater than the PhA on the left side [18]. Additionally, no segmental or whole-body measures of PhA were reported; therefore, the relationship between whole-body PhA and PhA derived from a single side of the body was not examined.

If BIA is to continue emerging as a clinical tool for assessing older adults, there remains a need to determine the relationship between whole-body and segmental BIA variables using modern BIA devices that use electrodes on both sides of the body. Understanding how to calculate whole-body PhA when only segmental values are reported may reduce the need for clinicians to replace their modern BIA devices with newer models. Therefore, the purpose of this investigation was to determine the relationship between whole-body PhA at 50 kHz generated from a modern BIA device (PhA_DG_) and whole-body PhA calculated at 50 kHz using the sum of segmental Z, Xc, and R (PhA_Calc_) from the left side, right side, and combined sides of the body in community-dwelling older adults. Additionally, we sought to compare both individual and summed BIA variables between the left and right sides of the body at 50 kHz.

## 2. Materials and Methods

### 2.1. Participants

**Figure 1:**
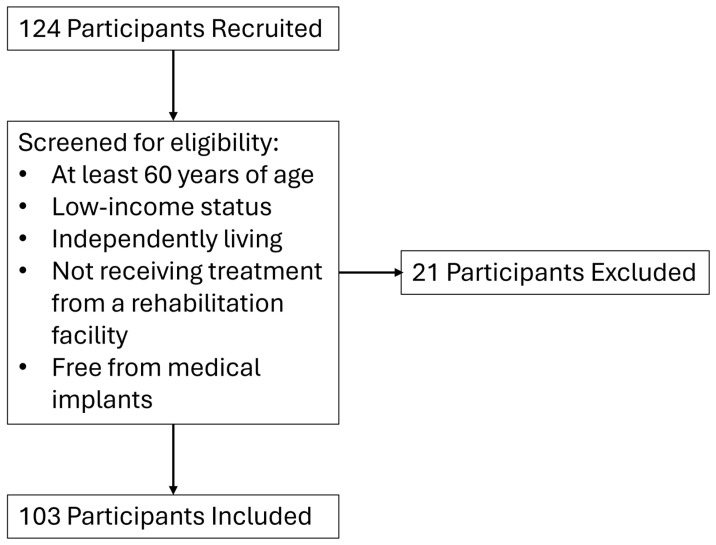
The flow of participants through recruitment, screening, and inclusion in this study.

This investigation was part of a federally funded research study (NIH Grant R03AG06799), for which the research protocol and methods have previously been reported [19]. A total of 103 community-dwelling older women (*n* = 87) and men (*n* = 16) participated in this cross-sectional investigation. All study procedures were approved by the University of Central Florida Institutional Review Board (IRB# STUDY00002189), pre-registered on ClinicalTrials.gov (NCT06063187), and carried out in accordance with the Declaration of Helsinki. All participants gave written informed consent prior to participation in this study. This study was conducted in community centers situated in low-income settings throughout the greater Orlando, FL, USA, metropolitan area. Figure 1 outlines the flow of participants in this study. Participants were recruited via flyer distribution, face-to-face engagement, local newsletters, and word of mouth. Community partners and clinical sites facilitated the introduction to potential participants for informed consent, initial screening, and eligibility verification using a checklist.

Participants were included if they were (i) aged ≥60 years, (ii) had low-income status based on 2019 United States poverty thresholds relative to family size and the number of children aged ≤18 years [20], (iii) and lived independently in their own homes or apartments. Those receiving active treatment from a rehabilitation facility and those with pacemakers were excluded from this study.

### 2.2. Bioelectrical Impedance Analysis

**Figure 2:**
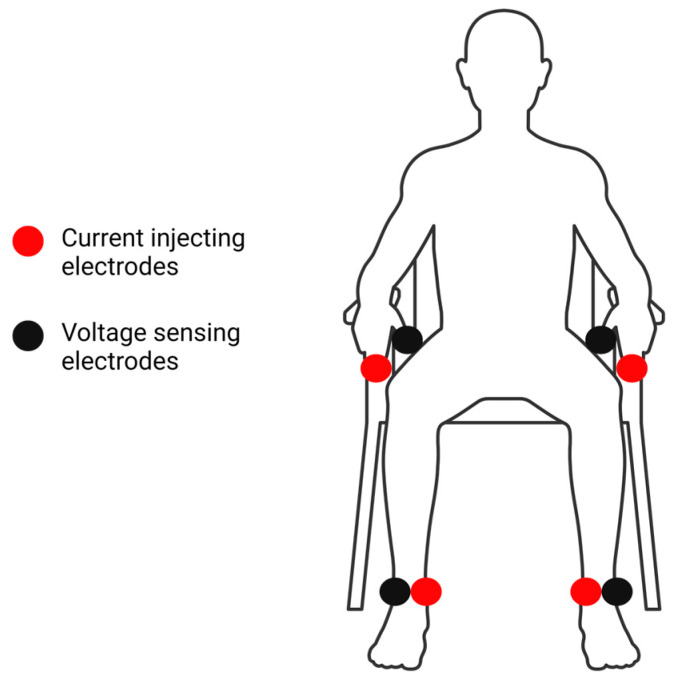
Locations of the InBody s10 touch-type electrodes in the seated position. Current-injecting electrodes were placed on the middle fingers and immediately inferior to the medial malleoli. Voltage-sensing electrodes were placed on the thumbs and immediately inferior to the lateral malleoli. Created with BioRender.com.

PhA was assessed using direct segmental multifrequency BIA with an InBody s10 device (Biospace, Seoul, Republic of Korea). The device measures Z at 1, 5, 50, 250, 500, and 1000 kHz and reports these values for individual body segments. Xc is measured at 5, 50, and 250 kHz and reported for individual body segments as well. R is not reported by the InBody s10 but, given the equation for Z (Z^2^ = R^2^ + Xc^2^) and its alignment with the Pythagorean Theorem, R can be considered the square-rooted difference between Z^2^ and Xc^2^ [21,22]. The InBody s10 calculates segmental and whole-body PhA at 50 kHz utilizing a proprietary equation. Prior to testing, participants were instructed to fast for 3–4 h, avoid caffeine and alcohol for 24 h, and abstain from exercise for 6–12 h prior to testing. Before testing, participants removed their socks, shoes, and any metal objects. Height and weight were assessed using a digital physician scale and stadiometer (Health-O-MeterTM, Model 402KL, McCook, IL, USA). Participants were then seated, and their skin was wiped with an InBody Tissue (Biospace, Seoul, Republic of Korea) at each electrode site. Figure 2 shows the placement of the electrodes. Touch-type electrodes were placed on the left and right ankles (inferior to the medial and lateral malleoli), middle fingers, and thumbs. The testing protocol required participants to remain motionless in their seated position for approximately one minute before removing the electrodes. The InBody s10 has demonstrated high test–retest reliability in older adult women [23].

### 2.3. Phase Angle Calculations

PhA was assessed and calculated at 50 kHz. For segmental PhA, the R and Xc for each respective body segment (left leg, right leg, left arm, right arm, trunk) were used with the known mathematical expression for PhA: [arc tangent (Xc/R) × (180/π)]. To determine whole-body PhA_Calc_ on the right and left sides, R and Xc were summed between respective arms and legs, as well as the trunk, in line with previous research [14]. To determine whole-body PhA_Calc_ from combined sides, R and Xc were summed from both arms, both legs, and the trunk. Moreover, the InBody s10 does not distinguish a left and right trunk, so the same trunk values were used for all calculations. Whole-body measurements of Xc, Z, and R were not reported by the InBody s10 and, therefore, were not compared with calculations.

### 2.4. Statistical Analysis

All statistical analyses were conducted using jamovi version 2.4.1 [24,25], and all data were stored and managed in a REDCap database hosted by the University of Central Florida [26,27]. A Kolmogorov–Smirnov test revealed that data were non-normal and, thus, nonparametric tests were used. Empirical median 2.5th and 97.5th quantiles were determined as the lower and upper nonparametric limits of agreement between PhA_DG_ and each PhA_Calc_ [28]. Relationships between whole-body PhA_Calc_ and PhA_DG_ values were assessed using Spearman’s rho (r_s_) coefficients, and comparisons were made using Wilcoxon rank and rank biserial correlation (r_rb_) effect sizes, which are interpreted similarly as r_s_ [29]. The sum of the bioelectric values on the right and left side were also compared using Wilcoxon rank. Data are presented as mean ± standard deviation where indicated to allow for easier comparison with previous research. Median and interquartile range values are also reported where indicated to account for non-normality. The threshold for statistical significance was set at *p* < 0.05.

## 3. Results

### 3.1. Participant Characteristics

Table 1 describes the participants included in the analyses. One male participant, an outlier with a PhA_DG_ greater than three standard deviations away from the mean, was excluded from the analysis. Thus, a total of 102 participants were included in the analyses. 

### 3.2. Whole-Body PhA

Table 2 outlines the results of the correlation matrix and nonparametric limits of agreement analysis used to assess the relationship of PhA_Calc_ with PhA_DG_ at the whole-body level.There was a statistically significant mean difference of 0.11° between PhA_Calc_ from the right and left sides with a standard error of 0.07° (*p* = 0.01, r_rb_ = −0.30). There was a statistically significant mean difference of 0.11° between PhA_Calc_ from the right and combined sides with a standard error of 0.07° (*p* < 0.001, r_rb_ = −0.56). PhA_Calc_ was not significantly different between the left and combined sides with a mean difference of 0.002° and a standard error of 0.14° (*p* = 0.91, r_rb_ = −0.01).

### 3.3. Segmental BIA Variables

Z, R, and Xc values for the right arm, right leg, and trunk were summed and compared to the sum of the left arm, left leg, and trunk’s Z, R, and Xc. Table 3 displays the results of both correlations and paired sample comparisons. There were significant differences between the summed left and right Z, R, and PhA_Calc_ values (*p* = 0.01), but the effect sizes were small to moderate. The right side had an average summed Z of 554.60 ± 94.52 Ω, an R of 552.02 ± 94.23 Ω, and an Xc of 52.06 ± 10.17 Ω. The left side had an average summed Z of 559.66 ± 99.55 Ω, an R of 556.80 ± 99.52 Ω, and an Xc of 53.25 ± 14.07 Ω.

Table 4 lists the comparison of means between left and right limbs. The left arm had an average Z of 345.45 ± 60.01 Ω, an R of 343.56 ± 60.17 Ω, an Xc of 33.88 ± 11.45 Ω, and a PhA_DG_ of 5.72 ± 2.15°. The right arm had an average Z of 340.42 ± 54.99 Ω, an R of 338.81 ± 54.88 Ω, an Xc of 32.64 ± 6.08 Ω, and a PhA_DG_ 5.54 ± 0.84°. The left leg had an average Z of 196.32 ± 48.04 Ω, an R of 195.58 ± 47.89 Ω, an Xc of 16.66 ± 5.08 Ω, and a PhA_DG_ of 4.87 ± 1.03°. The right leg had an average Z of 196.29 ± 47.44 Ω, an R of 195.55 ± 47.30 Ω, an Xc of 16.70 ± 4.89 Ω, and a PhA_DG_ of 4.90 ± 0.99°. All segmental PhA_Calc_ values were well correlated with the corresponding PhA_DG_ values with Spearman’s r_s_ coefficients ranging from −0.98 to −0.99 (*p* < 0.001). The lowest correlation was observed in the trunk (r_s_ = 0.98).

## 4. Discussion

The purpose of this study was to compare whole-body and segmental PhA_DG_ at 50 kHz to PhA_Calc_ from the left, right, and combined sides of the body among community-dwelling older adults. Our secondary aim was to compare individual and summed bioelectrical variables at 50 kHz from the right and left sides of the body. Whole-body PhA_DG_ was significantly different than PhA_Calc_ from the left side and combined sides, but not the right side. Furthermore, whole-body PhA_DG_ did not correlate well with left-sided PhA_Calc_ (r_s_ = 0.69; Table 2). This was in line with our hypothesis, as right-sided PhA_Calc_ demonstrated greater agreement with PhA_DG_. Bracco et al. reported nearly identical whole-body PhA_DG_ (9.8 ± 1.1°) and right-sided PhA_Calc_ (9.7 ± 1.5°) at 50 kHz [14]. In the present study, a similar relationship was observed with a small mean difference between whole-body PhA_DG_ and right-sided PhA_Calc_ (−0.004°; Table 2), which was likely due to rounding and may not represent a clinically significant difference given that PhA is most commonly reported to the tenths or hundredths decimal place [22]. Whole-body PhA_Calc_ from the left side and combined sides had greater and significant mean differences (0.11°) compared to PhA_DG_ (Table 2) as well as lower correlation coefficients, suggesting that PhA_Calc_ from the right side would be the most interchangeable with PhA_DG_.

Previous research has demonstrated a tendency for whole-body PhA assessed from the left side of the body to be lower than whole-body PhA assessed from the right side [18]. Previous comparisons between whole-body and left-sided Z have also demonstrated poorer agreement than right-sided Z [14,30,31], which may have also impacted the observed agreement between PhA_DG_ and PhA_Calc_ from combined sides, as it included left-sided Z. Strong correlations were observed between summed left- and right-sided Z, R, and Xc (r_s_ = 0.84–0.95, *p* < 0.001; Table 3), yet there was a significant difference in Z and R between sides of the body (*p* = 0.01, r_rb_ = −0.32; Table 3). Given that R and Z are factors in determining PhA, the observed difference between right- and left-sided PhA_Calc_ (*p* = 0.01, r_rb_ = 0.30) could be considered a consequence of differences in R and Z between sides. While the primary purpose of the present study was to compare calculated PhA to PhA reported by the BIA device at 50 kHz, these differences between sides of the body may be explained by confounding variables on the left side of the body and should be explored by future research.

One plausible confounding variable may be lean body mass distribution, as the hydration status and bioelectrical properties of lean body mass differ from those of fat mass [32]. Previous research has shown an asymmetrical distribution of muscle mass based on the side of the body that is predominantly used to produce force [33]. While the InBody s10 estimates lean body mass, those variables were not included in this study because the device has been shown to have poor reliability in estimating lean body mass in older adults compared to dual-energy X-ray absorptiometry [23]. Furthermore, lean body mass measures derived from BIA are based on the same Z, R, and Xc variables that lean body mass may confound, making it an inappropriate method in this context. Although the potential differences in lean mass distribution may explain differences in bioelectrical variables between lateral sides of the body, it was not controlled for in the present study, as this study primarily aimed to compare PhA_Calc_ to PhA_DG_, and BIA devices do not account for lean mass distribution/hand dominance when determining whole-body measures. This is also apparent in the use of electrodes on only the right side of the body with older BIA devices, regardless of hand dominance or lean mass distribution [14].

The heart may also be a plausible explanation for the difference between the bioelectric values of the left and right sides, as it is positioned predominantly on the left side of the body and carries its own conduction system. The electrical current delivered by BIA devices is thought to interfere with the conduction system of the heart and is a sufficiently low frequency for detecting the tissue structure of cardiac fibers [34]. Other abdominothoracic organs, such as the spleen and liver, anatomically bias one side of the body and serve as a reservoir of fluid that may be detected by the electrical current [35]. However, the InBody s10 reports one value for the trunk, which was used in both left- and right-sided PhA_Calc_. This suggests that any potential confounding factor affecting R, Z, and PhA on the left side would likely manifest in the left arm and/or leg. A comparison of BIA variables between the left and right arms revealed significant differences in Z, R, and PhA_DG_, while the legs showed no significant differences. The left arm had greater Z and R (Table 4), indicating greater opposition to the electrical current. However, this investigation aimed to determine whether differences existed between sides of the body rather than distinguishing causality, so more research is needed in that regard.

The present results differed from Jensen et al., as they reported no significant differences in PhA_Calc_ between sides [18]. These contrasting results may be due to differences in population and posture, as Jensen et al. assessed PhA standing and supine in adults and children [18]. In the present study, we were limited to only assessing in the seated position, so more research is needed in different postures. We were unable to measure lean body mass with a reliable instrument and did not assess handedness amongst participants, which limits the ability of the present results to determine causality for the observed differences. Furthermore, the InBody s10 does not report whole-body values of Z, R, and Xc, so no comparisons between calculated and device-generated whole-body values could be made for these variables. However, PhA encompasses Z, R, and Xc and may indicate that similar differences would exist. With PhA gaining clinical utility as an indicator of sarcopenia, diabetes, physical function, and other medical conditions [5,11,22,36], it is important that clinicians understand how to calculate whole-body PhA when only segmental Z, R, and Xc are reported by their BIA device. The present study provides evidence supporting calculations of whole-body PhA using right-sided bioelectrical variables in older adults. Future studies should explore the proposed and other confounding variables to determine why bioelectrical variables differ between sides of the body. Furthermore, more information is needed regarding how BIA devices measure whole-body bioelectrical variables when electrodes are placed on both sides of the body and if any estimations or corrections are employed.

## 5. Conclusions

At 50 kHz with a modern BIA device, right-sided PhA_Calc_ demonstrated superior agreement with PhA_DG_ at the whole-body level compared to PhA_Calc_ from the left and combined sides of the body, which is consistent with prior research that utilized older BIA devices with electrodes on a single side of the body. Z, R, and PhA differed between sides of the body and specifically between arms, indicating that the bilateral sides of the body are not interchangeable when determining whole-body bioelectrical measurements. With PhA increasing as a diagnostic marker of sarcopenia and frailty, measurement accuracy is crucial, and calculations based on segmental values may be necessary if BIA devices do not report whole-body Z, R, and Xc. Any calculation of whole-body bioelectrical variables should be based on measurements from a single side of the body, with the present data indicating that the right side would be the most appropriate choice.

## Figures and Tables

**Table 1:** Participant characteristics (N = 102).

	Age (Years)	Whole-Body PhA_DG_^1^ (°)	Body Mass Index (kg/m^2^)	Body Weight (kg)
Mean	75.7	5.4	26.9	73.2
Median	75.0	5.4	26.1	73.0
Standard deviation	7.2	0.9	5.2	15.8
Interquartile range	9.8	1.0	6.2	23.3
Minimum	60.0	3.0	18.2	42.2
Maximum	96.0	6.9	47.9	126.6

^1^ PhA_DG_ = device-generated PhA. Whole-body PhA_DG_ was measured at 50 kHz. PhA values are listed as degrees (°) measured at 50 kHz; PhA_DG_ = device-generated phase angle; PhA_Calc_ = calculated phase angle; SD = standard deviation; IQR = interquartile range; r_s_ = Spearman’s rho correlation coefficient; effect sizes are presented as rank biserial correlation (r_rb_).

**Table 2:** Comparison of whole-body phase angles (N = 102).

			Agreement						
Method	Mean ± SD	Median (IQR)	2.5th Quantile	97.5th Quantile	r_s_	Mean Difference	95% Confidence Interval	*p*	Effect Size
PhA_DG_	5.38 ± 0.80	5.40 (1.0)	0.002	0.05	0.97	−0.004	(−0.011, 0.003)	0.26	−0.13
Right Side PhA_Calc_	5.41 ± 0.75	5.40 (1.0)							
PhA_DG_	5.38 ± 0.80	5.40 (1.0)	0.02	1.88	0.69	0.11	(0.023, 0.174)	0.01	0.29
Left Side PhA_Calc_	5.51 ± 1.50	5.40 (1.1)							
PhA_DG_	5.38 ± 0.80	5.40 (1.0)	0.02	0.91	0.79	0.11	(0.067, 0.141)	<0.001	0.54
Combined Sides PhA_Calc_	5.40 ± 0.91	5.36 (1.0)							

**Table 3:** Comparison of left and right summed variables (N = 102).

	Correlation		Mean Differences				
Variable	r_s_	*p*	Wilcoxon W	Mean Difference	SE	*p*	Effect Size
Impedance (Z)	0.95	<0.001	1780	−5.05	2.75	0.01	−0.32
Resistance (R)	0.95	<0.001	1780	−4.78	2.82	0.01	−0.32
Reactance (Xc)	0.84	<0.001	2794	0.40	1.08	0.27	−0.13
PhA_Calc_	0.75	<0.001	3425	0.11	0.14	0.01	0.30

Z, R, and Xc values are listed as ohms (Ω) measured at 50 kHz; PhA values are listed as degrees (°); “Variable” represents the sum of a right arm, leg, and trunk compared to the sum of a left arm, leg, and trunk; r_s_ = Spearman’s rho correlation coefficient; SE = standard error; effect sizes are presented as rank biserial correlation (r_rb_).

**Table 4:** Comparison of left and right limbs (N = 102).

	Correlation		Mean Differences				
Variable	r_s_	*p*	Wilcoxon W	Mean Difference	SE	*p*	Effect Size
Arms							
Impedance (Z)	0.92	<0.001	1535	−6.70	2.27	<0.001	−0.42
Resistance (R)	0.91	<0.001	1532	−6.77	2.35	<0.001	−0.42
Reactance (Xc)	0.77	<0.001	2937	0.35	1.08	0.22	0.14
PhA_DG_	0.69	<0.001	1662	−0.19	0.24	0.001	−0.37
Legs							
Impedance (Z)	0.96	<0.001	2582	−0.20	1.17	0.88	−0.02
Resistance (R)	0.96	<0.001	2577	−0.24	1.17	0.87	−0.02
Reactance (Xc)	0.94	<0.001	2649	0.06	0.17	0.67	0.05
PhA_DG_	0.88	<0.001	2552	−0.004	0.06	0.81	−0.03

Z, R, and Xc values are listed as ohms (Ω) measured at 50 kHz; PhA values are listed as degrees (°); “Variable” represents the comparison of a left and right limb; r_s_ = Spearman’s rho correlation coefficient; SE = standard error; effect sizes are presented as rank biserial correlation (r_rb_).

## Data Availability

The data presented in this study are available upon request from the corresponding author, K.L. (ethical reasons).
